# Femoral fixation methods for hamstring graft in anterior cruciate ligament reconstruction: A network meta-analysis of controlled clinical trials

**DOI:** 10.1371/journal.pone.0275097

**Published:** 2022-09-22

**Authors:** Shixin Nie, Shuqing Zhou, Wei Huang

**Affiliations:** 1 Department of Orthopedics, The First Affiliated Hospital of Chongqing Medical University, Chongqing, China; 2 Orthopedic Laboratory of Chongqing Medical University, Chongqing, China; 3 Department of Orthopedics, The Centre Hospital of Jiangjin, Chongqing, China; Assiut University Faculty of Medicine, EGYPT

## Abstract

**Objective:**

To compare the clinical effectiveness of cortical button (CB), cross-pin (CP) and compression with interference screws (IS) fixation techniques in anterior cruciate ligament (ACL) reconstruction using hamstring graft.

**Methods:**

Studies were systematically retrieved from PubMed, Embase, Cochrane Library and Web of Science up to May 20, 2021. Primary outcomes were KT-1000 assessment, International Knee Documentation Committee (IKDC) score A or B, Lachman’s test, pivot-shift test, visual analogue scale (VAS) score, Lysholm score, Tegner score, and Cincinnati Knee Score. Secondary outcomes included reconstruction failures and synovitis. League tables, rank probabilities and forest plots were drawn for efficacy comparison.

**Results:**

Twenty-six controlled clinical trials (CCTs) with 1,824 patients undergoing ACL reconstruction with hamstring graft were included. No significant differences were found among CB, CP and IS fixation methods regarding the 10 outcomes. For KT-1000 assessment, IKDC score A or B, Lachman’s test, VAS score and pivot-shift test, CP had the greatest probability of becoming the best method, and IS may be the suboptimal method in 4 out of these 5 outcomes except pivot-shift test.

**Conclusions:**

CP, CB and IS fixations have comparable clinical performance, while CP fixation is most likely to be the optimum fixation technique for hamstring graft in ACL reconstruction. Future larger-sample studies of high quality comparing these techniques in more clinical outcomes are required.

## Introduction

Anterior cruciate ligament (ACL) rupture is a common knee ligament injury, which occurs more in the physically active population than in the general population [1]. This ACL injury can lead to pain, functional limitations, osteoarthritis after knee trauma, and reduced quality of life [2, 3]. In the United States, around 400,000 ACL reconstructions are carried out yearly [4]. Autologous hamstring graft is in widespread use and considered as the gold standard of ACL reconstruction, minimizing donor site morbidity [5–8]. However, hamstring graft movement within the femoral tunnel may impede tendon-to-bone healing, so having stable fixation is of great concern [9, 10].

Currently, femoral fixation methods for ACL reconstruction cover three categories: cortical button (CB) fixation, cross-pin (CP) fixation, compression with interference screws (IS) [11–13]. As for an optimal fixation technique, Ibrahim *et al*. [14] proposed that CP femoral fixation brought greater knee laxity outcomes than CB fixation, while CB femoral fixation exhibited similar effects to CP fixation concerning clinical outcomes and postoperative knee laxity in autologous hamstring ACL reconstruction according to a meta-analysis of Jiang *et al* [15]. CP fixation was shown to have a smaller instrumented side-to-side anterior-posterior laxity difference than IS fixation, but these two techniques demonstrated comparable performance for hamstring autograft [12]. In Björkman *et al*.’s research, femoral fixation with CP and IS provided a similar clinical or radiographic result in ACL reconstruction [16]. Additionally, CB fixation was superior to IS fixation for double-bundle ACL reconstruction [17], whereas equivalent impacts were obtained with IS and CB fixation in regard to knee anteroposterior stability and other aspects for all-inside ACL allograft reconstruction [18]. Unfortunately, no studies have reported direct comparisons among CB, CP and IS fixation measures for ACL reconstruction with hamstring graft, and which technique is the best remains unclear. Although a network meta‑analysis from Yan *et al*. [19] revealed that IS femoral fixation may be the most preferred approach in ACL reconstruction, including different types of studies may lower the statistical power in this study. Thus, a latest network meta‑analysis is needed to further probe into the optimum fixation method.

This study aimed to explore a superior femoral fixation method by comparing the efficacy of CB, CP and IS techniques via a network meta‑analysis of controlled clinical trials (CCTs) in ACL reconstruction with hamstring graft, which may serve as a reference in choosing a fixation method for better rehabilitation.

## Methods

### Search strategy and study selection

Studies concerning fixation methods in ACL reconstruction were systematically retrieved from PubMed, Embase, Cochrane Library and Web of Science up to May 20, 2021 by two investigators (SX Nie, SQ Zhou) independently. Search terms consisted of “Anterior Cruciate Ligament” OR “Anterior Cruciate Ligament Reconstruction” OR “Anterior Cruciate Ligament Injuries” OR “Anterior Cruciate Ligaments” OR “Cruciate Ligament, Anterior” OR “Cruciate Ligaments, Anterior” OR “Ligament, Anterior Cruciate” OR “Ligaments, Anterior Cruciate” OR “ACL” AND “Surgical Fixation Devices” OR “Orthopedic Fixation Devices” OR “Device, Fixation” OR “Devices, Fixation” OR “Fixation Device” OR “Fixation Devices” OR “Fasteners” OR “Fastener” OR “Fixator” OR “Fixators” OR “Bone Screws” OR “Screw” OR “Screws” OR “TransFix” OR “Intrafix” OR “Aperfix” OR “Arthrex” OR “Biotransfix” OR “Endobutton” OR “Rigidfix”. Then these studies were imported into Endnote X9 (Clarivate Analytics, Philadelphia, Pennsylvania, USA) for duplicate removal, and preliminary screening based on titles and abstracts was carried out, followed by full-text screening, so as to obtain qualified studies. Discussion was needed when opinions were divided.

### Inclusion and exclusion criteria

Inclusion criteria were: (1) studies with patients undergoing ACL reconstruction with hamstring graft; (2) studies with interventions including ≥ 2 femoral fixation techniques; (3) studies exploring at least one of the following outcomes; (4) studies in English; (5) CCTs.

The interventions were divided into three categories: (1) CB (Endobutton, Ligament Anchor, Swing Bridge, Tightrope) fixation; (2) CP (Intrafix, Transfix, Rigid Fix, aperture fixation) fixation; (3) IS (Metal Interference Screw, Bioabsorbable Interference Screw).

Exclusion criteria were: (1) animal experiments; (2) publications that did not meet the research theme; (3) studies where valid data could not be extracted; (4) conference abstracts, case reports, editorial materials, reviews, and meta-analyses.

### Outcome measures

Primary outcomes were KT-1000 (MEDmetric Corp, San Diego, CA, USA) assessment, International Knee Documentation Committee (IKDC) score A or B [20], Lachman’s test [21], pivot-shift test [22], visual analogue scale (VAS) score [23], Lysholm score [24], Tegner score [25], and Cincinnati Knee Score [26]. Secondary outcomes included reconstruction failures and synovitis.

### Data extraction

Two independent researchers (SX Nie, SQ Zhou) extracted baseline information from the eligible studies. The information included author, year of publication, country, level of evidence, femoral tunnel placing, femoral fixation, graft type, tibial fixation, sample size, age, gender ratio, time from injury to surgery, follow-up time, and outcome measure. A consensus was reached through discussion with a third researcher (W Huang).

### Risk of bias assessment

The risk of bias in each included CCT was evaluated applying the Cochrane Collaboration’s tool [27] by two reviewers separately (SX Nie, SQ Zhou). The domains for assessment included random sequence generation, allocation concealment, blinding of participants and personnel, blinding of outcome assessment, incomplete outcome data, selective reporting, and other bias. The risk of bias was categorized as low, unclear or high. Disagreements were resolved by a third researcher (W Huang).

### Quality of evidence assessment

The quality of evidence in pairwise effect estimates and overall ranking of femoral fixation methods was evaluated with the approach proposed by Salanti *et al*. [28] which was based on methodology developed by the Grading of Recommendations Assessment, Development and Evaluation (GRADE) Working Groups. Five domains were assessed: study limitations, indirectness, inconsistency, imprecision, and publication bias. Then the quality of evidence was divided into four levels: high, moderate, low and very low.

### Statistical analysis

R 4.0.3 software (R Foundation for Statistical Computing, Vienna, Austria) was employed for the network meta-analysis, and conventional meta-analysis was conducted with Stata 15.1 software (Stata, College Station, Texas, USA). Odds ratios (ORs) acted as the effect size of categorical outcomes, and standardized mean differences (SMDs) were used as the effect size of continuous outcomes. All estimates of these effect sizes reported were posterior medians with corresponding 95% credibility intervals (CrIs). When 95% CrIs excluded null values, significant effects of the femoral fixation methods on the different outcomes were identified. For each outcome measure, both fixed effects model and random effects model were initially fitted. Four Markov chains were adopted for every model to set initial values. The number of pre-iterations was set to 40,000, and the number of iteration operations was set to 200,000. The final model of each outcome was confirmed for subsequent analysis to attain the relative effects and ranking probabilities of different fixation measures in each outcome. In addition, the network plot, league table, rank probabilities and forest plot of each outcome measure were drawn. Node-split analysis was performed for consistency and inconsistency detection in direct and indirect comparisons when there was a closed loop. The strength of direct and indirect evidence was consistent if the difference between the deviance information criteria (DIC) of the consistency and inconsistency detection results was less than 5. *P* < 0.05 indicated a statistically significant difference.

## Results

### Characteristics of the included studies

**Fig 1:**
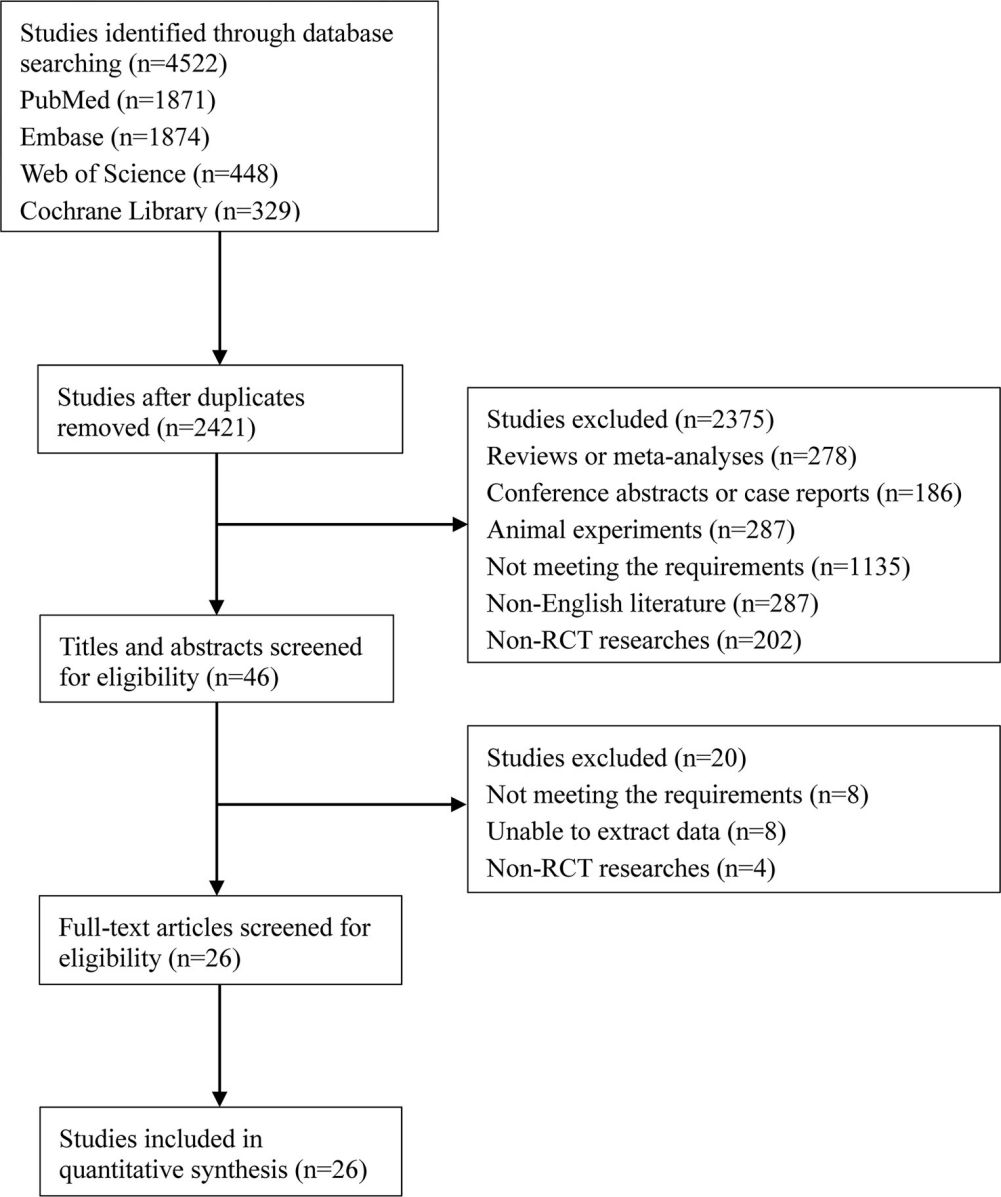
Flow chart for search process.

**Fig 2:**
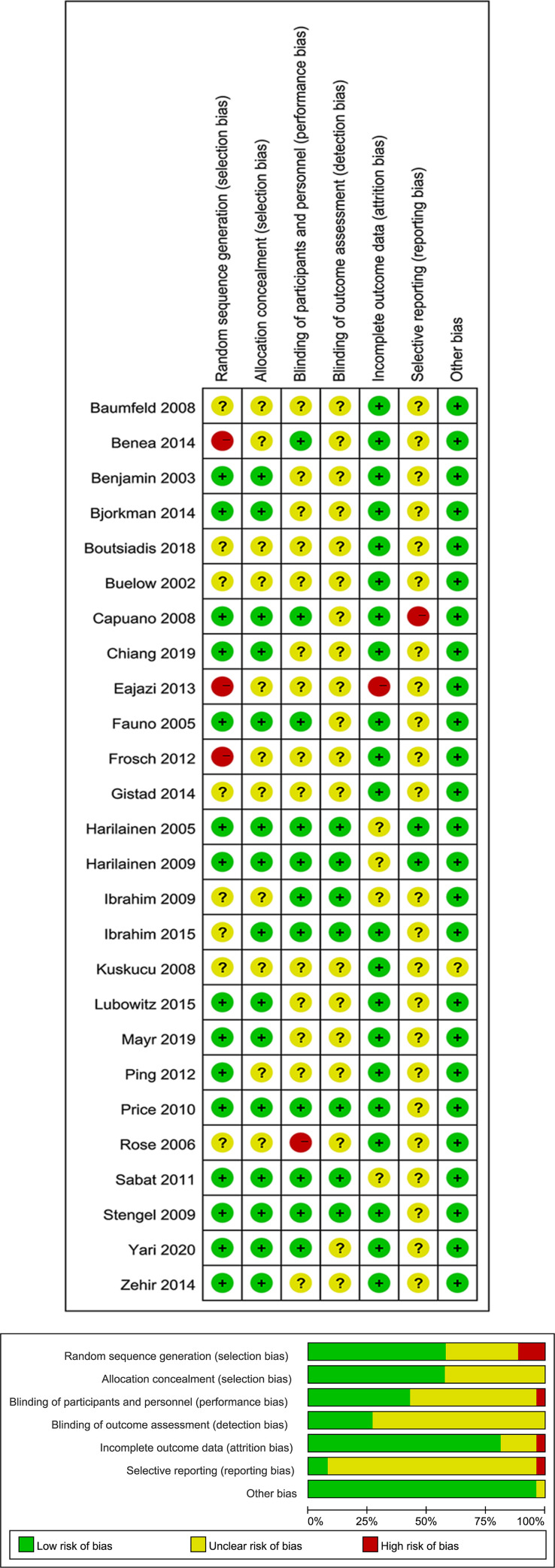
Risk of bias assessment for the included studies.

Based on the search strategy, 4,522 studies were identified from the four databases. After duplicates were removed, 2,421 studies were left. Following that, screening was carried out by reading titles and abstracts, and then full texts. Finally, 26 CCTs [14, 16, 18, 29–51] with 1,824 patients were qualified for next analysis. Detailed search process is illustrated in Fig 1. These included trials were published between 2002 and 2020, with 9 trials of CB vs CP [14, 29, 36, 37, 42, 43, 46, 48, 51], 9 of CB vs IS [18, 30–33, 35, 44, 45, 50], and 8 of CP vs IS [16, 34, 38–41, 47, 49]. Follow-up time ranged from 6 months to 60 months. Table 1 exhibits the baseline data of the included trials. The major risk of bias was selection bias from random sequence generation. The overall risk of bias of these studies was low. Risk of bias assessment for the qualified studies is shown in Fig 2. The quality of evidence in pairwise effect estimates ranged from very low to high, and most of pairwise comparisons had high quality of evidence. The overall ranking of femoral fixation methods for KT-1000 assessment, IKDC score A or B, Lachman’ s test, Pivot-shift test, and VAS score had moderate, high, high, high, and low quality of evidence, respectively (Table 2).

**Table 1:** Baseline characteristics of the included studies.

Author	Year	Country	Level of evidence	Femoral tunnel placing	Femoral fixation	Graft type	Tibial fixation	Sample size	Age, years	Sex (male/female)	Time from injury to surgery	Follow-up, months	Outcome measure
**CB vs CP**													
Fauno	2005	Denmark	I		CB	STG	PLLA IS	46	25	19/27	NA	12	KT-1000 assessment, IKDC score A/B
					CP		Bi IS/SW	41	26	19/22	NA		
Kuskucu	2008	Turkey	II	Transtibial-femoral drilling	CB	STG	IS and a staple	24	23.9 (21–44)	0/24	2–8 m	26.7 (16–36)	Lysholm score, IKDC score A/B, Tegner score
					CP			32		0/32		25.2 (12–36)	
Baumfeld	2008	USA	II	Transtibial drilling	CB	STG	Intrafx	26	35.9±12.0	NA	NA	41.8±13.4	KT-1000 assessment, IKDC score A/B, reconstruction failures
					CP		Bio IS	20	36.2±11.8			45.2±12.6	
Ibrahim	2009	Kuwait	I	Transtibial drilling	CB	SB and DB STG	NA	98	(22–33)	NA	2–3.7 m	29 (25–38)	Pivot-shift test, Lysholm score, IKDC score A/B
					CP	SB STG		102	(21–31)		2–4 m		
Price	2010	Australia	I	Transtibial drilling	CB	STG	Bio IS	11	26.5 (16–47)	NA	NA	24	Lachman’s test, IKDC score A/B
					CP			13	26.3 (16–48)				
Sabat	2011	India	II	Transtibial drilling	CB	STG	Bio IS	30	(20–40)	NA	6 w-2 y	12	Lysholm score, IKDC score
					CP								
Eajazi	2013	Iran	II		CB	SB STG	IS	33	26.2 (18–44)	NA	14.5 (2–80) m	24	Lysholm score, reconstruction failures
					CP			29	23.6 (19–31)		14.1 (1–84) m		
Zehir	2014	Turkey	II	Transtibial drilling	CB	STG	Bio IS	67	NA	NA	13.17±8.22 m	12	Lysholm score, IKDC score A/B, Tegner score, KT-1000 assessment, pivot-shift test
					CP			51			9.74±4.12 m		
Ibrahim	2015	Kuwait	II	Transtibial drilling	CB	DB STG	BioIntraFix	32	(22–32)	NA	2–4.2 m	30	Lachman’s test, pivot-shift test, KT-1000 assessment, Lysholm score, IKDC score A/B
					CP			34	(21–34)		2–4.5 m		
**CB vs IS**													
Buelow	2002	Australia	II		CB	STG	Bio IS	28	30.9 (17–44)	NA	NA	24	KT-1000 assessment, IKDC score A/B, Cincinnati Knee Score
					IS			30	30.9 (17–44)	17/13			
Benjamin	2003	USA	II		CB	STG	IS	15	22±10	3/12	NA	39±8 (24–50)	IKDC scores, KT differences
					IS			15	27±8	4/11		32±6 (24–40)	
Ping	2012	China	II		CB	DB STG	Bio IS	28	24.3 (18–38)	17/11	NA	29.5 (12–46)	Lachman’s test, pivot-shift test
					IS			35	25.5 (17–40)	22/13		28.5 (12–48)	
Benea	2014	France	I		CB	ST/STG	SutureButton	22	29.3±9	NA	25.7±46 m	6	VAS, IKDC score A/B
					IS			22					
Lubowitz	2015	USA	II		CB	STG	Arthrex	21	40.2±11.9	11/20	NA	24	IKDC score A/B
					IS			22	41.6±9.1	9/18			
Boutsiadis	2018	France	Ⅲ		CB	STG	IS	151	31.0±10.8	89/62	3.7±1.6 m	25.8±4.3	IKDC score A/B, pivot-shift test
					IS			121	32.6±10.6	64/57	3.4±1.5 m	25.6±2.3	
Chiang	2019	China	II		CB	DB STG	Cortical screw	28	29.5±5.7	26/2	NA	24	IKDC score, KT-1000 assessment, pivot-shift test
					IS			29	30.3±6.9	28/1			
Mayr	2019	Austria	II		CB	STG	IS	16	25±6	11/5	12 m	24	IKDC score A/B, pivot-shift test
					IS			14	29±7	10/4			
Yari	2020	USA	I		CB	STG	Bio IS	17	37.7±5.3	8/9	NA	6	VAS, IKDC score
					IS			16	36.9±6.7	9/7			
**CP vs IS**													
Harilainen	2005	Finland	I		CP	SB STG	Metal IS	26	27 (15–56)	NA	6 m (3 w-13 y)	24	Lachman’s test, pivot-shift test, KT-1000 assessment, IKDC score A/B
					IS			30	32 (28–49)		10 m (4 w-27 y)		
Rose	2006	Germany	I	Transtibial drilling	CP	STG	Bone Plug	38	28.5 (15–47)	22/16	NA	12	IKDC score A/B
					IS		Delta Screw	30	25.5 (13–61)	20/10			
Capuano	2008	France	I		CP	ST/STG	Milagro	15	30.6±9.8 (15–52)	10/5	16.9±14.7 (1–60) m	13.1±2.45	IKDC score A/B
					IS		IS	15	32.3±9.5 (15–49)	10/5	20.4±22.9 (1–74) m		
Harilainen (1)	2009	Finland	I	Transtibial drilling	CP	DB STG	BioScrew/IntraFix	28	31 (18–50)		4 m (1 w-10 m)	24	IKDC score A/B
					IS			29	35 (20–48)		3.5 m (1 w-35 m)		
Harilainen (2)					CP			25	29 (18–50)		4 m (1 w-32 m)		IKDC score A/B
					IS			25	32 (18–49)		3 m (1 w-8.25 y)		
Stengel	2009	Germany	I	Transtibial-femoral drilling	CP	ST/STG	RigidFix	24	31.4±12.2	NA	NA	24	KT-1000 assessment, IKDC scores, synovitis
					IS		Bio IS	21	26.1±10.4				
Frosch	2012	Germany	II		CP	ST/STG	Milagro IS	28	28.2 ±8.0	18/10	11.09±4.0 w	12.40±0.8	Tegner score, KT-1000 assessment, VAS
					IS			31	24.6 ±7.2	19/12	14.91±3.4 w	12.45±1.1	
Bjorkman	2014	Finland	I	Transtibial drilling	CP	SB STG	AO Screw/SW	25	NA	NA	NA	60	Lachman’s test, pivot-shift test
					IS	SB ST/STG		22					
Gifstad	2014	Norway	II	Transtibial drilling	CP	STG	WasherLoc	47	24 (18–45)	NA	≥ 6 w	24	KT-1000 assessment
					IS			46					

CB: cortical button; CP: cross-pin; IS: interference screw; SB: single bundle; DB: double bundle; ST: semitendinosus; STG: semitendinosus and gracilis; SW: spiked washer; w: week; m: months; y: years; IKDC: International Knee Documentation Committee; VAS: visual analogue scale.

**Table 2:** Summary of our confidence in effect estimates and ranking of femoral fixation methods.

Outcomes	Comparison	Nature of the evidence	Confidence	Downgrading due to
KT-1000 assessment	CB vs CP	Mixed	High	-
	CB vs IS	Indirect	Low	Study limitations^1^; Indirectness^2^
	CP vs IS	Mixed	Moderate	Study limitations^1^
	Ranking of treatments		Moderate	Study limitations^5^
IKDC score A or B	CB vs CP	Mixed	High	-
	CB vs IS	Mixed	Moderate	Imprecision^4^
	CP vs IS	Mixed	High	-
	Ranking of treatments		High	-
Lachman’ s test	CB vs CP	Mixed	High	-
	CB vs IS	Mixed	Low	Imprecision^4^; Inconsistency^3^
	CP vs IS	Mixed	High	-
	Ranking of treatments		High	-
Pivot-shift test	CB vs CP	Mixed	High	-
	CB vs IS	Mixed	Low	Study limitations^1^; Inconsistency^3^
	CP vs IS	Mixed	High	-
	Ranking of treatments		High	-
VAS score	CB vs CP	Indirect	Very low	Study limitations^1^; Imprecision^4^; Indirectness^2^
	CB vs IS	Mixed	Moderate	Inconsistency^3^
	CP vs IS	Mixed	Low	Study limitations^1^; Imprecision^4^
	Ranking of treatments		Low	Study limitations^1^; Imprecision^4^

^1^Dominated by evidence at high or moderate risk of bias.

^2^No convincing evidence for the plausibility of the transitivity assumption.

^3^Predictive intervals for treatment effect include effects that would have different interpretations (there is additionally no convincing evidence for the plausibility of the transitivity assumption).

^4^Confidence intervals include values favoring either treatment.

^5^60% of the information is from studies at moderate risk of bias.

IKDC: International Knee Documentation Committee; VAS: visual analogue scale; CB: cortical button; CP: cross-pin; IS: interference screw.

### Network plots of fixation method comparisons

**Fig 3:**
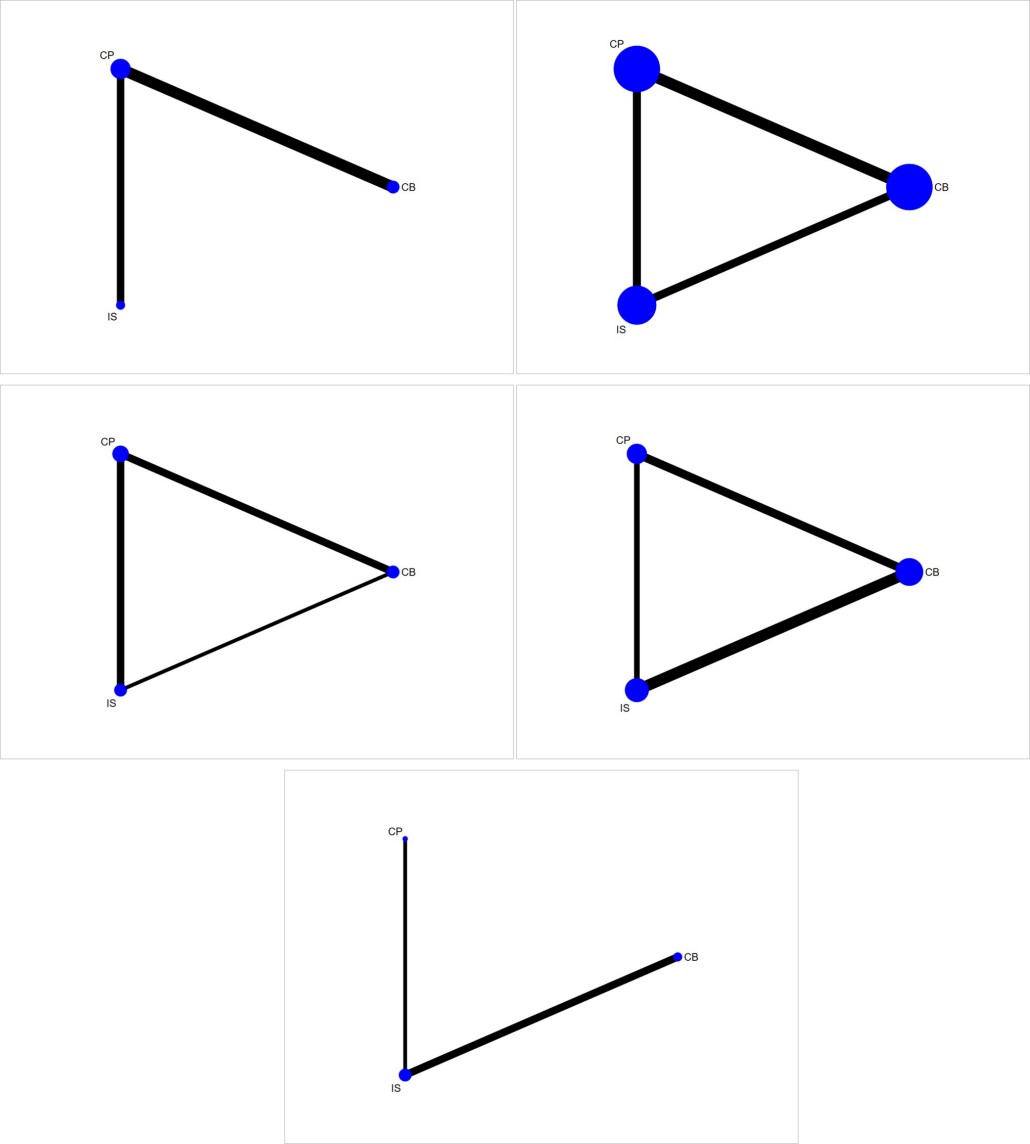
Network plots of fixation method comparisons for various outcomes. 3A: KT-1000 assessment; 3B: IKDC score A or B; 3C: Lachman’s test; 3D: Pivot-shift test; 3E: VAS score. IKDC: International Knee Documentation Committee; VAS: visual analogue scale; CB: cortical button; CP: cross-pin; IS: interference screw.

Network plots were depicted to reflect comparisons among CB, CP and IS fixation techniques (Fig 3). Five studies provided data for KT-1000 assessment. Direct comparisons were available between CP and IS, and between CP and CB, but there was no direct comparison between IS and CB. Most studies were performed on CP, followed by CB; direct comparison evidence for CP and CB was most abundant. As to IKDC score A or B, 16 CCTs were included. Direct evidence on the pairwise comparison of CP, CB and IS was displayed, constituting a closed-loop relationship. Most studies were done on both CP and CB, and on the direct comparison of these two methods. A closed loop was also formed for CP, CB and IS in terms of Lachman’s test which was explored in 5 studies. CP fixation was reported in the majority of the 5 studies, and most evidence for direct comparison was offered on CP and IS. Pivot-shift test was conducted in 9 trials. With the direct pairwise comparison of the three techniques, a closed-loop relation came into being. Most of the studies focused on CB, and on the head-to-head comparison of CB and IS. Besides, 3 trials were conducted on VAS score. Direct comparisons existed between CP and IS, and between IS and CB. Most trials investigated IS, followed by CB. Most direct evidence of comparison was available for IS and CB. According to the above, closed loops were formed for IKDC score A or B, Lachman’s test and pivot-shift test, and the results of node-split analysis indicated that the strength of the direct and indirect evidence was consistent (Table 3).

**Table 3:** Consistency and inconsistency detection for the outcomes.

Outcomes	DIC for consistency detection	DIC for inconsistency detection	Absolute value of ΔDIC
IKDC score A or B	49.190	49.138	0.052
Lachman’ s test	13.889	13.938	0.049
Pivot-shift test	13.946	13.969	0.023

IKDC: International Knee Documentation Committee; DIC: deviance information criteria; ΔDIC: difference between the DIC of the consistency and inconsistency detection results.

### League tables for fixation methods

The efficacies of CP, CB and IS techniques on different outcomes were compared in pairs, as shown in Table 4. As regards KT-1000 assessment, no significant differences were observed between CP and CB (pooled OR = 3.725, 95% CrI = 0.407–55.092), between IS and CB (pooled OR = 2.054, 95% CrI = 0.031–113.409), and between IS and CP (pooled OR = 0.551, 95% CrI = 0.015–10.848). Likewise, the effectivity of these 3 methods were similar in other 4 outcomes: IKDC score A or B (CP vs CB: pooled OR = 1.838, 95% CrI = 0.868–3.743; IS vs CB: pooled OR = 1.554, 95% CrI = 0.673–3.781; IS vs CP: pooled OR = 0.841, 95% CrI = 0.380–2.102), Lachman’s test (CP vs CB: pooled OR = 1.511, 95% CrI = 0.523–4.406; IS vs CB: pooled OR = 1.153, 95% CrI = 0.324–4.092; IS vs CP: pooled OR = 0.758, 95% CrI = 0.284–2.104), pivot-shift test (CP vs CB: pooled OR = 1.254, 95% CrI = 0.577–3.203; IS vs CB: pooled OR = 0.564, 95% CrI = 0.253–1.582; IS vs CP: pooled OR = 0.456, 95% CrI = 0.159–1.357), and VAS score (CP vs CB: pooled SMD = 1.135, 95% CrI = -3.438–6.773; IS vs CB: pooled SMD = 0.862, 95% CrI = -1.829–4.541; IS vs CP: pooled SMD = -0.298, 95% CrI = -4.298–3.715).

**Table 4:** League tables of fixation methods for various outcomes.

KT-1000 assessment	CB	3.725 (0.407, 55.092)	2.054 (0.031, 113.409)
	1.315 (-0.898, 4.009)	CP	0.551 (0.015, 10.848)
	0.720 (-3.485, 4.731)	0.596 (-2.384, 4.205)	IS
IKDC score A or B	CB	1.838 (0.868, 3.743)	1.554 (0.673, 3.781)
	-0.609 (-1.320, 0.142)	CP	0.841 (0.380, 2.102)
	-0.441 (-1.330, 0.396)	0.173 (-0.743, 0.969)	IS
Lachman’s test	CB	1.511 (0.523, 4.406)	1.153 (0.324, 4.092)
	-0.413 (-1.483, 0.648)	CP	0.758 (0.284, 2.104)
	-0.142 (-1.409, 1.127)	0.277 (-0.744, 1.258)	IS
Pivot-shift test	CB	1.254 (0.577, 3.203)	0.564 (0.253, 1.582)
	-0.226 (-1.164, 0.550)	CP	0.456 (0.159, 1.357)
	0.574 (-0.458, 1.373)	0.786 (-0.305, 1.838)	IS
VAS score	CB	1.135 (-3.438, 6.773)	0.862 (-1.829, 4.541)
	-1.135 (-6.773, 3.438)	CP	-0.298 (-4.298, 3.715)
	-0.862 (-4.541, 1.829)	0.298 (-3.715, 4.298)	IS

IKDC: International Knee Documentation Committee; VAS: visual analogue scale; CB: cortical button; CP: cross-pin; IS: interference screw.

### Rank probabilities for fixation methods

Rank probabilities were illustrated for CP, CB and IS fixation approaches (Tables 5–9). As for KT-1000 assessment, IKDC score A or B, Lachman’s test, VAS score and pivot-shift test, CP had the greatest probability of becoming the best method, and IS may be the suboptimal method in 4 out of 5 outcomes except pivot-shift test.

**Table 5:** Rank probabilities of fixation methods for KT-1000 assessment.

	[1]	[2]	[3]
CB	0.088500	0.285750	0.625750
CP	0.581900	0.380725	0.037375
IS	0.329600	0.333525	0.336875

CB: cortical button; CP: cross-pin; IS: interference screw.

**Table 6:** Rank probabilities of fixation methods for IKDC score A or B.

	[1]	[2]	[3]
CB	0.022513	0.153988	0.823500
CP	0.653313	0.314863	0.031825
IS	0.324175	0.531150	0.144675

IKDC: International Knee Documentation Committee; CB: cortical button; CP: cross-pin; IS: interference screw.

**Table 7:** Rank probabilities of fixation methods for Lachman’s test.

	[1]	[2]	[3]
CB	0.173313	0.287325	0.539363
CP	0.583563	0.329688	0.086750
IS	0.243125	0.382988	0.373888

CB: cortical button; CP: cross-pin; IS: interference screw.

**Table 8:** Rank probabilities of fixation methods for pivot-shift test.

	[1]	[2]	[3]
CB	0.257175	0.650113	0.092713
CP	0.705275	0.249663	0.045063
IS	0.037550	0.100225	0.862225

CB: cortical button; CP: cross-pin; IS: interference screw.

**Table 9:** Rank probabilities of fixation methods for VAS score.

	[1]	[2]	[3]
CB	0.124785	0.203350	0.671865
CP	0.563155	0.235190	0.201655
IS	0.312060	0.561460	0.126480

VAS: visual analogue scale; CB: cortical button; CP: cross-pin; IS: interference screw.

### Forest plots for fixation methods

According to the results of forest plots, CP and CB (CP vs CB: pooled OR = 3.800, 95% CrI = 0.410–58.000) as well as IS and CP (IS vs CP: pooled OR = 0.520, 95% CrI = 0.014–10.000) exhibited comparable effects concerning KT-1000 assessment. For IKDC score A or B, no statistically significant differences existed between CP and CB (CP vs CB: pooled OR = 2.200, 95% CrI = 0.930–5.900), IS and CB (IS vs CB: pooled OR = 1.000, 95% CrI = 0.280–3.500), and IS and CP (IS vs CP: pooled OR = 1.200, 95% CrI = 0.440–6.200). Consistently, the equivalent effectiveness of CP and CB (CP vs CB: pooled OR = 1.600, 95% CrI = 0.490–5.700), IS and CB (IS vs CB: pooled OR = 0.760, 95% CrI = 0.076–5.900), and IS and CP (IS vs CP: pooled OR = 0.830, 95% CrI = 0.280–2.400) was demonstrated in Lachman’s test. CP and CB (CP vs CB: pooled OR = 1.200, 95% CrI = 0.390–3.300), IS and CB (IS vs CB: pooled OR = 0.670, 95% CrI = 0.250–2.700), and IS and CP (IS vs CP: pooled OR = 0.310, 95% CrI = 0.036–1.700) also had similar impacts on pivot-shift test. In terms of VAS score, IS did not significantly differ from CB (IS vs CB: pooled SMD = 0.860, 95% CrI = -1.800–4.500) and CP (IS vs CP: pooled SMD = -0.300, 95% CrI = -4.300–3.700). Three studies reported on Lysholm score, and combined analysis revealed that CB presented similar efficacy to CP (CB vs CP: pooled OR = 1.220, 95% confidence interval (CI) = 0.460–3.240, *P* = 0.686). When reconstruction failures were taken into consideration, the comprehensive analysis of 2 studies indicated that there was no statistical difference between CB and CP (CB vs CP: pooled OR = 1.010, 95% CI = 0.390–2.670, *P* = 0.977).

### Fixation methods for single or double bundle ACL reconstruction

For KT-1000 assessment, only one study compared CP and IS fixation methods in single bundle ACL reconstruction, and no significant difference was found between CP and IS (OR = 0.833, 95% CI = 0.211–3.294, *P* = 0.795); there was no report on double bundle ACL reconstruction. Regarding IKDC score A or B, CP was shown to have a similar effect to IS in single bundle ACL reconstruction according to a single study (OR = 2.000, 95% CI = 0.525–7.621, *P* = 0.310); network meta-analysis was performed for double bundle ACL reconstruction based on 2 studies, and revealed that IS was significantly more effective than CB (pooled OR = 1.307, 95% CrI = 3.695, 62.929) and CP (pooled OR = 1.180, 95% CrI = 3.254–54.762), and CP was likely to be better than CB according to rank probabilities. Concerning Lachman’s test, 2 studies provided direct evidence for the comparison between CP and IS in single bundle ACL reconstruction, and meta-analysis demonstrated no significant difference between CP and IS (pooled OR = 1.175, 95% CI = 0.463–2.986, *P* = 0.734). Network meta-analysis with data from 2 trials for double bundle ACL reconstruction illustrated that CP, CB and IS had comparable impacts on Lachman’s test, and rank probabilities indicated that CP had the highest probability of becoming the optimal method (64.77% probability), and CB was most likely to be the suboptimal method (52.89% probability). As to pivot-shift test, direct evidence from 2 studies on the comparison between CP and IS in single bundle ACL reconstruction exhibited equivalent effectiveness of CP and IS (pooled OR = 2.645, 95% CI = 0.637–10.984, *P* = 0.181); for double bundle ACL reconstruction, network meta-analysis of 3 trials showed no significant difference among CP, CB and IS, while CP was most likely to be the best fixation method (77.41% probability), and CB was most likely to be the second best method (53.75% probability). Besides, no studies about VAS score reported the technique used for ACL reconstruction (single bundle or double bundle).

### Fixation methods when placing the femoral tunnel via transtibial drilling

Twelve studies reported the technique used for placing the femoral tunnel, and all of them used transtibial drilling. Among these 12 studies, 3 had KT-1000 assessment, and network meta-analysis exhibited that CP, CB and IS had similar influences on KT-1000 assessment, while CP was most likely to be the optimum fixation method (47.60% probability), and IS was most likely to be the suboptimum method (35.52% probability). Concerning IKDC score A or B, 8 trials provided data for comparisons among CP, CB and IS. It was found that IS was significantly more effective than CB (pooled OR = 1.323, 95% CrI = 1.005–3.732), and IS had the greatest probability of becoming the best method (93.35% probability), and the second best method was most likely CP (85.49% probability). As regards Lachman’s test, 3 studies were included for network meta-analysis. No significant differences were observed among CP, CB and IS; IS was most likely to be the optimal method (53.31% probability), and CP was most likely to be the suboptimal method (51.90% probability). With respect to pivot-shift test, 4 studies were qualified. Consequently, IS was significantly less effective than CB (pooled OR = 0.001, 95% CrI = 4.887×10^−18^–0.464) and CP (pooled OR = 0.001, 95% CrI = 4.887×10^−18^–0.441) for pivot-shift test, and CP had the highest likelihood of being the best method (58.87% probability). Of the 12 studies, none assessed VAS score.

## Discussion

The current network meta-analysis found with 26 CCTs of 1,824 patients that CP, CB and IS displayed similar effects on different clinical outcomes in ACL reconstruction with hamstring graft, which was consistent with the findings of previous meta-analyses [52–54] and network meta-analyses [19, 55]. Nevertheless, CP may be more effective than CB and IS for hamstring graft fixation in ACL reconstruction according to rank probabilities analysis; based on this, CP may be prioritized in the femoral fixation of hamstring grafts for ACL reconstruction, so that more satisfactory recovery could be expected.

The development of CP for femoral fixation in ACL reconstruction intends to deal with underlying problems linked to IS and CB techniques, with less anteroposterior laxity and sufficient mechanical strength [56, 57]. An instrumented side-to-side anterior-posterior laxity difference was prominently reduced by CP versus IS, as reported by Hu and others [12]. This supports our revelation to a certain extent that CP had a higher probability of being better to IS in fixing hamstring graft for ACL reconstruction, in terms of knee stability, pain, function and physical activities. The possible superiority of CP to IS concerning knee stability may be due to the fact that CP fixation is performed strictly because anchorage looseness is not allowed, while for IS, the looseness relevant to the tunnel wall occurs [49]. A systematic review reported that the failure rates of bioabsorbable IS, metallic IS and CP were 6.1, 3.3 and 1.7%, separately [58], indicating that CP with a higher success rate can be selected before IS. Additionally, compared with CP femoral fixation, IS was associated with a markedly higher risk of ACL revision for patients receiving ACL reconstruction [59]. Furthermore, CP may be more efficacious than CB in femoral fixation for hamstring ACL reconstruction in this paper. A meta-analysis by Lee *et al*. [60] demonstrated more femoral tunnel widening after applying CB fixation than CP fixation to reconstruct ACL. CB fixation was also in association with more laxity compared with CP [30]. Tunnel widening possibly links to knee laxity and graft failure [61], and consequently requires a staged revision through bone grafting [62], which is not conducive to the recovery of patients suffering from torn ACL. The above findings reinforce the possibility of CP as the optimum fixation. Apart from the afore-mentioned studies [19, 52–55], the studies of Hu *et al* [12] and Jiang *et al* [15] also showed that IS and CB femoral fixations had equivalent impacts on clinical performance to CP femoral fixation for ACL reconstruction with hamstring graft. Similar effects of CP, IS and CB are confirmed using different analytical methods, and more investigations on the comparisons of these three techniques are necessary to validate that CP is most likely to be the best fixation technique.

Among the included studies, regarding Tegner score, Kuskucu *et al*. [43] discovered that among 24 patients receiving CB fixation, 17 improved from level 4 to level 6 or 7, and the other patients remained at level 4 or 5. After CP fixation, 25 of 32 patients improved from level 4 to level 6 or 7, and the rest of patients remained at level 4 or 5. The Tegner score in the CB and CP groups was reported by Zehir *et al*. [51] to be comparable. Frosch *et al*. [38] showed that the average Tegner score was 5.83 points (±2.00) for IS fixation and 5.83 points (±1.24) for CP fixation, and no significant difference was found between the two groups. Since the data from the above studies cannot be synthesized, we only described the results of these studies. Besides, merely Buelow *et al*. [33] studied the Cincinnati Knee Score of patients in the CB and IS groups during a 2-year follow-up period. The CB group increased from preoperative 44 ± 9.8 to 87 ± 8.9, and the IS group elevated from preoperative 46 ± 10.2 to 86 ± 8.5, without a significant difference between the two groups. Of note, Stengel *et al*. [49] reported 1 out of 28 patients undergoing CP fixation developed synovitis, and 4 out of 26 patients having IS fixation suffered from synovitis, suggesting that patients receiving CP fixation might have a lower rate of adverse events than those with IS fixation in ACL reconstruction. This necessitates more research into adverse event occurrences after the three fixation methods. Given the highest likelihood of CP femoral fixation being the optimal method in hamstring graft for ACL reconstruction as regards clinical efficacy, together with safety, surgeons may give priority to CP fixation when performing ACL reconstruction, combined with their experience and proficiency as well as the cost of surgery, so that patients could get better rehabilitation under their timely and effective decision-making.

With respect to the technique used for ACL reconstruction (single bundle or double bundle) and the technique used for placing the femoral tunnel (transtibial or transportal or outside-in), 8 studies reported the technique used for ACL reconstruction; 12 studies reported the technique used for placing the femoral tunnel, and all of them applied transtibial drilling. Based on the above information, we have assessed the effect of the femoral fixation methods on the outcomes under these reported ACL reconstruction and femoral tunnel placing techniques. For pivot-shift test under double bundle ACL reconstruction and KT-1000 assessment under the transtibial drilling technique, CP, CB and IS exerted similar influences, while CP was most likely to be the optimum fixation method, which was consistent with our main findings, indicating that these techniques might have no effects on pivot-shift test under double bundle ACL reconstruction and KT-1000 assessment under transtibial drilling. However, this is just a conjecture, and we cannot determine whether these techniques have effects on the outcomes, because most studies did not report on these techniques. Relevant studies should provide complete information on ACL reconstruction and femoral tunnel placing techniques, so that the impact of these techniques on the outcomes can be assessed and a better femoral fixation method can be offered to patients undergoing ACL reconstruction with hamstring graft for better recovery.

Through this network meta-analysis of CCTs, CP fixation was recommended as the first choice to fix hamstring grafts in ACL reconstruction. Nonetheless, certain limitations cannot be ignored. First, the included studies did not provide direct evidence for comparisons among CP, CB and IS on some outcome measures, and there was subjectivity in the outcome evaluation. Besides, this analysis could not determine whether the technique used for ACL reconstruction (single bundle or double bundle) and the technique used for placing the femoral tunnel (transtibial or transportal or outside-in) have effects on the outcomes, since most studies did not report on these techniques. Second, heterogeneity probably from different fixation devices, surgical methods and follow-up time was not addressed. Further, studies in other languages were not included. Third, for KT-1000 assessment, the comparison of CB and IS was based on indirect evidence of low confidence, and for VAS score, the comparison of CB and CP was based on indirect evidence of very low confidence, which may affect the reliability of the comparison results that IS may be better than CB for KT-1000 assessment and CP may be better than CB for VAS score. Future high-quality evidence is warranted to verify these results.

## Conclusion

CP, CB and IS fixations exhibit similar clinical performance, whereas CP fixation has the greatest probability of being more effective than CB and IS for hamstring graft in ACL reconstruction. This study underscores the need for further larger-sample studies of high quality to compare the impacts of these techniques on more clinical outcomes.

## Supporting information

S1 Checklist PRISMA 2020 checklist. (DOCX)
